# Supplementary material for: One-Step Calcination to Gain Exfoliated g-C3N4/MoO2 Composites for High-Performance Photocatalytic Hydrogen Evolution
Source: Molecules. 2022 Oct 24;27(21):7178. doi: 10.3390/molecules27217178 (PMC9658904; doi:10.3390/molecules27217178)
Supplement: Supplementary file 1 [file molecules-27-07178-s001.zip › molecules-1961426-supplementary.pdf]

# Supporting Information

## One-Step Calcination to Gain Exfoliated $g-C_3N_4/MoO_2$ Composites for High-Performance Photocatalytic Hydrogen Evolution

**Yan Chen [1,2], Ao Li [1,2], Xiuli Fu [2] and Zhijian Peng [1,*]**

[1] School of Science, China University of Geosciences, Beijing 100083, China
[2] School of Science, Beijing University of Posts and Telecommunications, Beijing 100876, China
[*] Correspondence: pengzhijian@cugb.edu.cn; Tel.: +86-10-82320255

This work was supported by the National Natural Science Foundation of China (grant nos. 11674035 and 61274015), and the Fundamental Research Funds for the Central Universities.

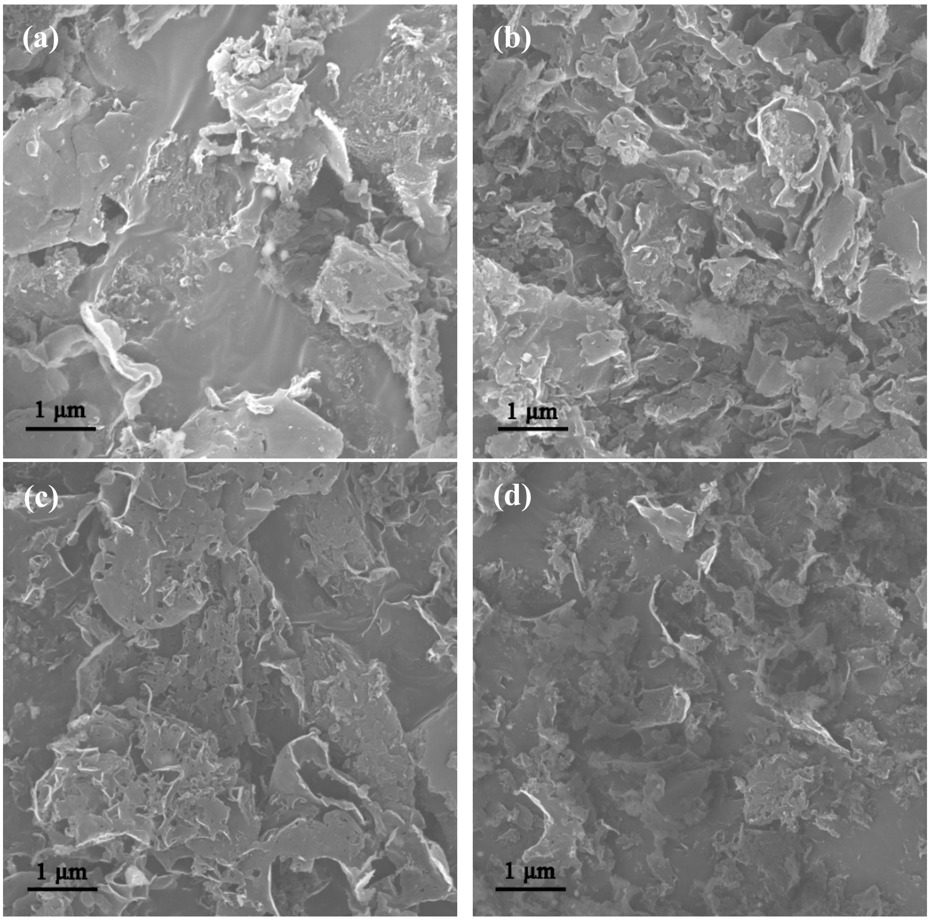


**Figure S1** Typical low-magnification SEM images of (a) CNM-1, (b) CNM-2, (c) CNM-4, (d) CNM-8.

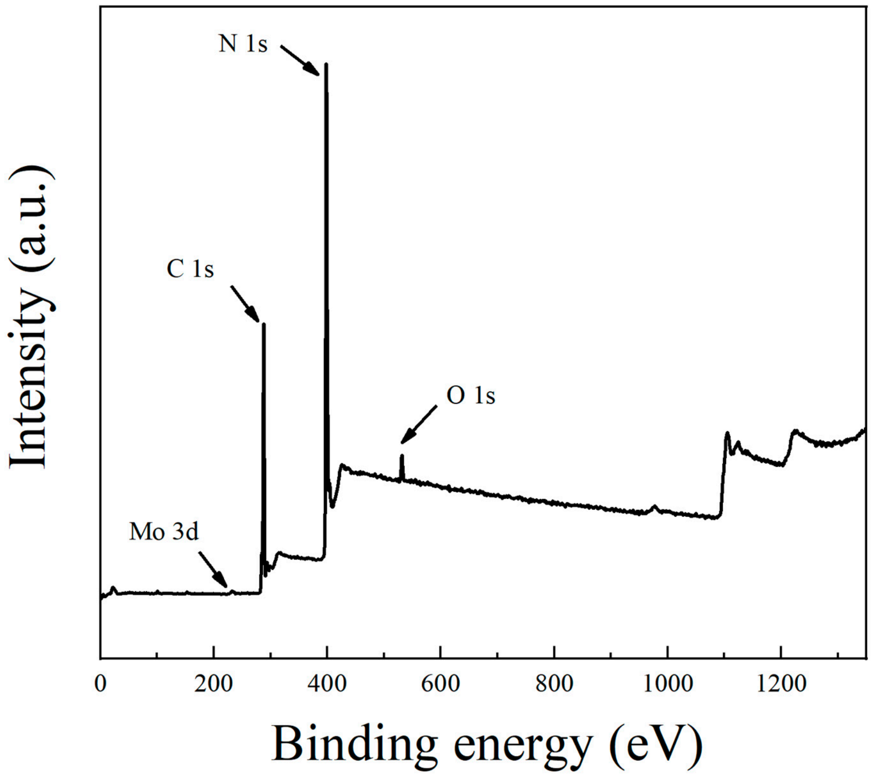


**Figure S2** XPS survey spectrum of the optimal sample CNM-4.

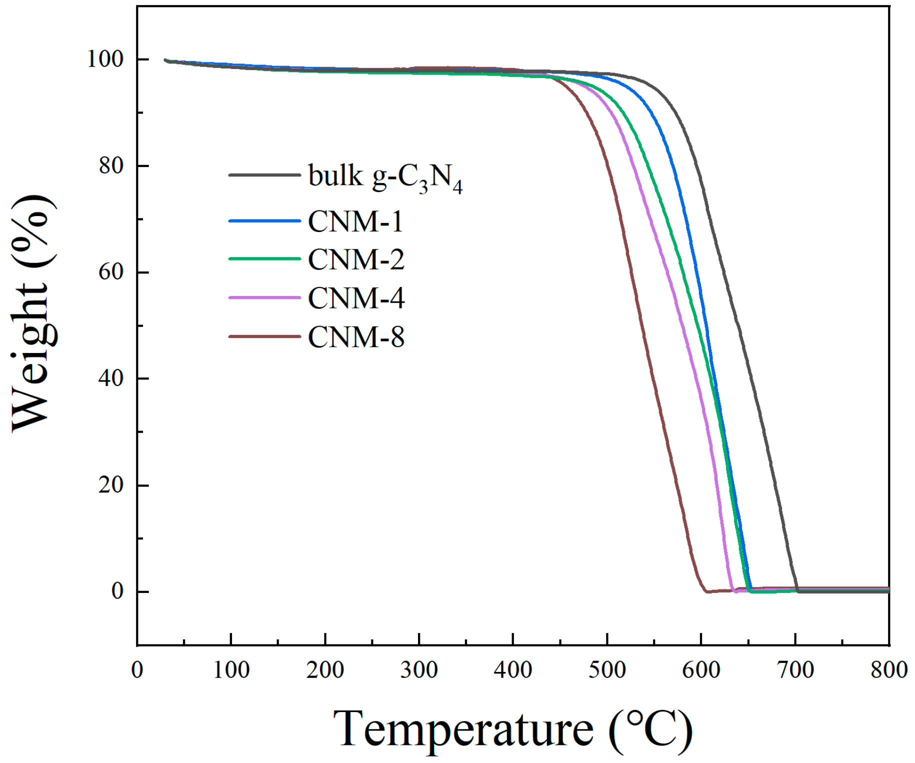


**Figure S3** TG curves of bulk g-$C_3N_4$ and the precursors to prepare the different exfoliated g-$C_3N_4$/$MoO_2$ composites.

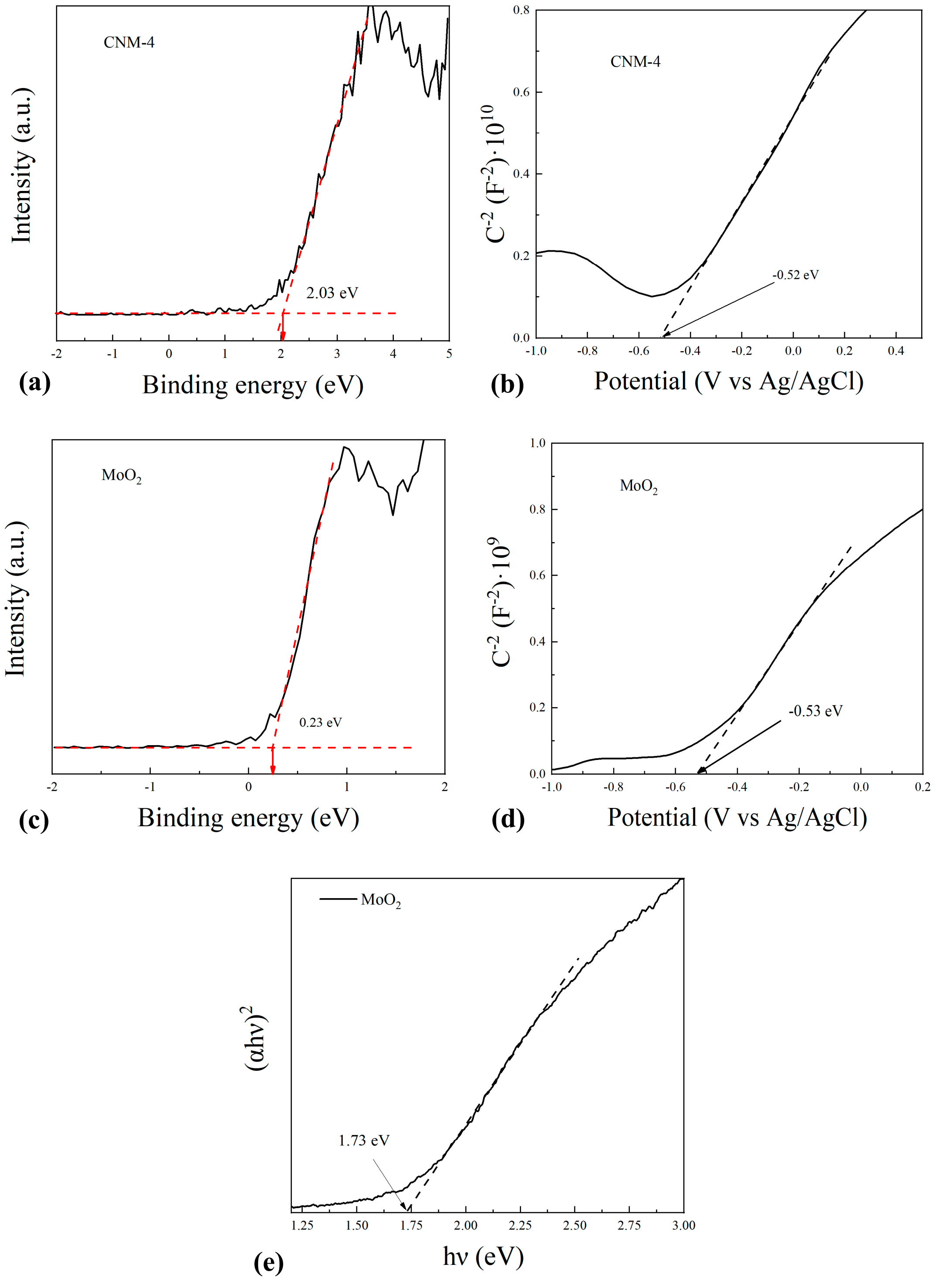


**Figure S4.** Valence-band XPS spectrum (a) and Motto-Schottky plot (b) of the optimal sample CNM-4. Valence-band XPS spectrum (c) and Motto-Schottky plot (d) of $MoO_2$. (e) $(\alpha h\nu)^2$ versus $E_g$ plot of $MoO_2$.
